# Integrating environmental conservation and public health strategies to combat zoonotic disease emergence: a call to action from the Amazon rainforest

**DOI:** 10.3389/fcimb.2024.1405472

**Published:** 2024-04-24

**Authors:** Esteban Ortiz-Prado, Justin Yeager, Jorge Vasconez-Gonzalez, Marco Culqui-Sánchez, Juan S. Izquierdo-Condoy

**Affiliations:** ^1^ One Health Research Group, Universidad de Las Americas, Quito, Ecuador; ^2^ Grupo de Investigación en Biodiversidad, Medio Ambiente y Salud (BIOMAS), Facultad de Ingenierías y Ciencas Aplicadas, Universidad de Las Américas, Quito, Ecuador; ^3^ Facultad de Ciencias de la Salud, Pontificia Universidad Católica del Ecuador, Quito, Ecuador

**Keywords:** zoonotic diseases, environmental conservation, public health, strategies, Amazon rainforest

Many species are influenced by the pressures derived from antagonistic co-evolutionary relationships, such as those between hosts and pathogens. Human civilizations in particular have experienced numerous, well-documented pandemics. With enhanced interconnectivity between what were once isolated populations, the frequency and scope of outbreaks or epidemics have since evolved into pandemics. The concept of “spillover” – the zoonotic transmission of viruses, bacteria, parasites, and even fungus (from animals to humans) is a central mechanism that explains the emergence, and reemergence, of many infectious diseases (Ellwanger and Chies, 2021). Historical instances such as influenza, rabies, and the recent catastrophic SARS-CoV-2 pandemic, which caused unprecedented global mortality and morbidity, underscore the critical nature of better addressing this issue (Thumbi et al., 2022; Chaves et al., 2023; Schumacher et al., 2024). Here we outline instances where deforestation and other environmental degradation practices have enhanced the frequency of zoonotic transmissions from wildlife to people. We additionally outline some best practices that can be applied throughout Latin America to mitigate the risk of transmission through the adoption of urgently needed changes to implement conservation measures and enforce environmental protection policies. Below, we detail specific concerns regarding zoonotic risks to urban and rural populations in South American countries, particularly those situated at the borders of tropical forests.

Mechanisms of infectious disease transmission such as spillover are intricately linked to cultural and environmental contexts, which are exemplified in culturally diverse and biodiverse ecosystems such as the Amazon rainforest. In the Amazonian region, countless reticulated interactions can be found between indigenous populations and flora and fauna. By hunting various species such as armadillos, which are carriers of leprosy (*Dasypus novemcinctus*), known to be naturally infected with *Mycobacterium leprae* and implicated in the zoonotic transmission of leprosy, rural human populations in the United States find themselves frequently in contact with a disease that has been largely eradicated in suburban and urban settings (White and Franco-Paredes, 2015). In light of the domestication of non-traditional pet species, including monkeys, turtles, and tortoises, which are known carriers of pathogens such as *Salmonella, Shigella*, and *Campylobacter*, there arises a clear and direct pathway for disease transmission (Silva et al., 2018; Keatts et al., 2021; Vale et al., 2021; Zúñiga, 2021; Wegner et al., 2022). The detailed examination of these diseases’ transmission mechanisms highlights the complex interactions between humans, animals, and their shared environments, especially in regions adjacent to tropical forests where zoonotic spillover risks are heightened. Therefore, it is imperative to develop and implement targeted strategies to mitigate the risk of future pandemics. This includes education in areas where cultural practices such as hunting and consuming wild animals prevail, aiming to train the population to minimize risks (**Figure 1**).

**Figure 1:**
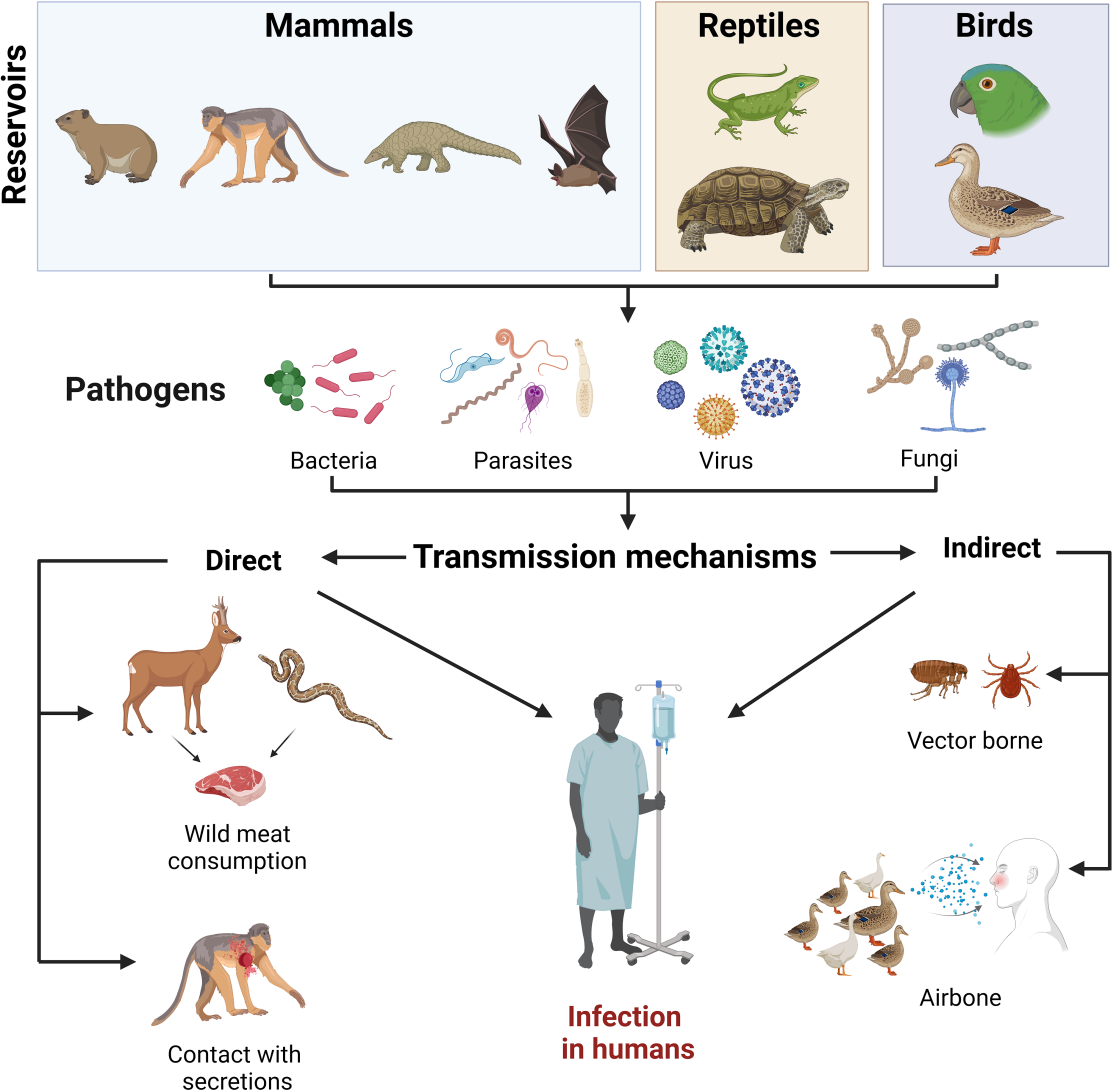
Illustration of a range of animal species and their interaction with humans, highlighting the practices of hunting and consumption that lead to direct or indirect contact. Similarly, the enforcement of wildlife laws which control the domestication of high-risk wildlife species, public education to raise awareness of zoonotic risks, and increased research to better understand disease dynamics are essential.

Similarly, the enforcement of wildlife laws which control the domestication of high-risk wildlife species, public education to raise awareness of zoonotic risks, and increased research to better understand disease dynamics are essential.

Although total mitigation of zoonotic disease risk is likely unattainable, by adopting the strategies we can attenuate unnecessary risks to rural and indigenous populations, and early detection sampling will aid with rapid containment. This is timely given zoonotic diseases account for 75% of all emerging infectious diseases, with a significant proportion originating from wildlife species (**Table 1**) (Gebreyes et al., 2014). The recent COVID-19 pandemic has highlighted risks associated with bushmeat consumption, calling for increased medical and research attention to the interplay between dietary practices associated with hunting non-domesticated species and the potential for zoonotic spillover (Milbank and Vira, 2022). To mitigate risks associated with zoonotic pathogens, a balanced approach is necessary that simultaneously safeguards public health, without undermining the dietary needs of communities dependent on non-domesticated food sources.

**Table 1:** Examples of key zoonotic diseases, their causative pathogens, and associated wild animal hosts, highlighting the critical role of wildlife in disease transmission and the need for targeted conservation and management efforts.

Disease	Pathogen type	Pathogen	Transmission	Wild Animal Host	Discovery of the pathogen
Chagas disease	Parasite	*Trypanosoma cruzi*	Through the feces of triatomines infected with the parasite	Opossums, rodents, armadillos, monkeys	1909
Trichinellosis	Parasite	*Trichinella* spp.	Eating raw or undercooked meat that contains the roundworm larvae	Bear, moose and wild boar	1835
Visceral larva migrans	Parasite	*Baylisascaris procyonis*, *Toxocara canis*, *Toxocara cati, and* *Ascaris suum*	Fecal-oral route	Birds, emus, cats, chinchillas, porcupines, prairie, rabbits, weasels, woodchucks, and woodrats, foxes, wolfs, coyotes	1952
Anthrax	Bacteria	*Bacillus anthracis*	Contact with infected animals through butchering and working with hides or ingestion of raw or undercooked meat	Elk, deer, mink	1876
Bubonic plague	Bacteria	*Yersinia pestis*	Bite from a flea	Squirrels, wood rats, prairie dogs, mice, voles, chipmunks, rabbits	1894
Leprosy	Bacteria	*Mycobacterium leprae*	Meat consumption	Monkeys, rats, mice, armadillo	1873
Rabies	Virus	*Rabies lyssavirus.*	Through direct contact with saliva or brain, nervous system tissue from an infected anima	Bats, monkeys, wolves, skunks, rabbits, coyotes	1882
Ebola	Virus	*Ebolavirus*	Contact with infected animals when preparing, cooking or eating them; body fluids of infected people	Monkeys, gorillas, chimpanzees, apes, wild antelopes	1976
Sporotrichosis	Fungi	*Sporothrix schenckii*	Contact with the fungal spores in the environment	Birds, rats, armadillos, stray cats	1898

Table adapted from (Rahman et al., 2020; Recht et al., 2020).

The degradation of the Amazon rainforest through deforestation, wildfires, and climate change has escalated the risk of the region becoming a hotspot for Emerging Infectious Diseases (EIDs). Deforestation, whether due to logging, mining, urbanization, or livestock activity, results in habitat fragmentation and can reduce available supplies of food and water. This in turn causes wildlife to migrate to other habitats that may be urbanized (Lennox et al., 2016). In addition, the loss of habitat causes the displacement of wildlife, which can increase the interaction of wildlife with domestic animals resulting in additional bridges for the circulation of pathogens between wild animals and humans (Ellwanger and Chies, 2018; Wilkinson et al., 2018). Mining, which has increased in frequency throughout the Amazon, rapidly degrades habitats and has specifically been associated with greater exposure to diseases such as malaria (Ellwanger et al., 2020; Martins et al., 2022). Increased proximity between humans and wildlife species (and their pathogens) facilitates the emergence/reemergence of zoonotic diseases.

Agriculture can shape pathogen persistence by merging wild and domestic animal habitats, increasing pathogen transfer risks. Changes in land use, irrigation, and crops can also alter disease vector populations, like mosquitoes and ticks, boosting disease transmission to animals and humans. Thus, agricultural practices significantly heighten pathogen spillover risks, highlighting the importance of integrated management strategies in agriculture to reduce these threats (Ellwanger et al., 2020). On top of that, the current trends on deforestation accompanied by an increase in temperature due to global climate change has allowed the expansion of vectors such as the *Aedes* mosquito, which facilitates the transmission of arboviruses responsible for Zika, Chikungunya, Dengue, Yellow fever, Oropouche (Thomson and Stanberry, 2022). The expansion of *Aedes* mosquito vectors is facilitated and enhanced by habitat degradation and climate change, underlying the need for synergistic multidisciplinary responses encompassing the medical field, public health, environmental sciences, and public policy sectors (Lowe et al., 2020).

The diversity of transmission routes and the potential for severe negative effects to human populations can be exemplified by viral hemorrhagic fevers (VHFs) caused by various RNA virus families, which are excellent cases to illustrate the complex ecological determinants of disease spread (Flórez-Álvarez et al., 2022). The experience of South America with diseases such as Argentinian, Brazilian, Venezuelan, Bolivian, and Chapare hemorrhagic fevers reinforces the vital importance of maintaining ecosystem health as a preventive strategy against zoonotic diseases (Frank et al., 2021). Humans can acquire the infection through inhalation of urine particles or feces from infected rodents or by direct contact through skin wounds (Frank et al., 2021). A recent review has afforded a detailed example using spillover from bats and detailing mechanistic reasons where zoonotic spillover could occur and methods to mitigate this risk (Plowright et al., 2024). Here we expand upon this work by detailing additional possible sources of zoonotic infections and highlight where the shortcoming of contemporary conservation attempts in the Amazon region are increasing the risk of spillover.

In light of the compelling evidence we outline, we consider it necessary to mobilize a unified global response that transcends traditional geographic and thematic boundaries between conservation and public health interests. This call-to-action urges researchers, policymakers, environmentalists, indigenous community leaders, and the broader global health community to foster collaborative efforts aimed at preserving the ecological integrity of the Amazon rainforest, and similar ecosystems, worldwide. There are five main policies aimed at the conservation of the Amazon. These involve establishing protected areas and indigenous reserves, promoting the use of organic products and responsible trade, reducing illegal activities by outsiders (including hunting, fishing, mining, and logging), enhancing sustainable biocommerce, and creating mechanisms for compensating ecosystem services (Killeen, 2023).

In 2023, the Belém Declaration was signed, which establishes a cooperation agenda on various fronts to ensure the preservation of the Amazon and promote the sustainable development of the region, which adds to existing treaties such as the Leticia Pact for the Amazon and the Amazon Cooperation Treaty (Rodriguez, 2023). While these initiatives are powerful contributions to the agenda we outline, they are incomplete solutions, and have been plagued by problems with their implementation due to problems such as corruption, and therefore have ultimately have lacked the capacity and resources to fully address and combat environmental crimes (Mistler-Ferguson, 2022).

We advocate for the implementation of robust anti-deforestation policies, the empowerment and respect of indigenous land rights, and the advancement of comprehensive surveillance and control measures for zoonotic diseases. Through a concerted multidisciplinary approach to conservation, we can attenuate chances of novel zoonotic transmission of diseases by reducing unnecessary contact with wildlife, reducing the risk of transmission from wildlife to domestic animals. Given recent renewed interests and concerns related to zoonotic diseases, action is timely.

In conclusion, the prevention of future pandemics necessitates a cohesive and informed response that addresses the root causes of zoonotic spillover, including environmental degradation and unsuitable interactions with wildlife. By fostering global cooperation and implementing targeted interventions, we can aspire to safeguard public health while preserving the ecological integrity of vital regions like the Amazon rainforest.

## Author contributions

EO: Conceptualization, Data curation, Formal analysis, Investigation, Methodology, Supervision, Validation, Visualization, Writing – original draft, Writing – review & editing. JY: Investigation, Software, Supervision, Validation, Writing – review & editing. JV: Data curation, Investigation, Methodology, Resources, Visualization, Writing – original draft. MC: Investigation, Methodology, Resources, Validation, Visualization, Writing – original draft. JI: Data curation, Investigation, Methodology, Project administration, Resources, Supervision, Validation, Visualization, Writing – original draft, Writing – review & editing.
